# Mechanisms of antibiotic resistance

**DOI:** 10.3389/fmicb.2015.00034

**Published:** 2015-02-05

**Authors:** Jun Lin, Kunihiko Nishino, Marilyn C. Roberts, Marcelo Tolmasky, Rustam I. Aminov, Lixin Zhang

**Affiliations:** ^1^Department of Animal Science, The University of TennesseeKnoxville, TN, USA; ^2^Institute of Scientific and Industrial Research, Osaka UniversityOsaka, Japan; ^3^Department of Environmental and Occupational Health Sciences, University of WashingtonSeatle, WA, USA; ^4^Center for Applied Biotechnology Studies, Department of Biological Science, California State UniversityFullerton, CA, USA; ^5^Section for Bacteriology, Pathology, and Parasitology, National Veterinary Institute, Technical University of DenmarkFrederiksberg, Denmark; ^6^CAS Key Laboratory of Pathogenic Microbiology and Immunology, Institute of Microbiology, Chinese Academy of SciencesBeijing, China

**Keywords:** antibiotics, resistance, mechanisms, resistance reservoirs, metagenomics, combination therapy

Antibiotics represent one of the most successful forms of therapy in medicine. But the efficiency of antibiotics is compromised by a growing number of antibiotic-resistant pathogens. Antibiotic resistance, which is implicated in elevated morbidity and mortality rates as well as in the increased treatment costs, is considered to be one of the major global public health threats (www.who.int/drugresistance/en/) and the magnitude of the problem recently prompted a number of international and national bodies to take actions to protect the public (http://ec.europa.eu/dgs/health_consumer/docs/road-map-amr_en.pdf: http://www.who.int/drugresistance/amr_global_action_plan/en/; http://www.whitehouse.gov/sites/default/files/docs/carb_national_strategy.pdf). Understanding the mechanisms by which bacteria successfully defend themselves against the antibiotic assault represent the main theme of this eBook published as a Research Topic in *Frontiers in Microbiology: Antimicrobials, Resistance, and Chemotherapy*. The articles in the eBook update the reader on various aspects and mechanisms of antibiotic resistance. A better understanding of these mechanisms should facilitate the development of means to potentiate the efficacy and increase the lifespan of antibiotics while minimizing the emergence of antibiotic resistance.

The multidrug efflux systems contribute significantly to the increased resistance to multiple antibiotics in bacteria. A major challenge in developing efficacious antibiotics against drug-resistant pathogens is to identify compounds that can counteract the efflux functions. The wealth of bacterial genomics information available suggests the presence of a variety of efflux systems in bacteria. Even a single bacterium may possess multiple efflux transporters of different families, with the overlapping substrate spectra. Accumulating evidence has indicated that the MexXY multidrug efflux system is a primary determinant of aminoglycoside resistance in *Pseudomonas aeruginosa*. Morita et al. (2012) provided a timely review on the *P. aeruginosa* MexXY pump and other aminoglycoside efflux pumps in a range of different bacteria. The expression of bacterial multidrug efflux system is usually controlled by transcriptional regulators that either repress or activate the transcription of the multidrug efflux genes. The articles by Usui et al. (2013) and Deng et al. (2013) further illustrated the complexity of regulation of multidrug efflux systems. However, the importance of multidrug efflux system may not be overstated for a specific antibiotic or organism, which is supported by the findings of Baucheron et al. (2014).

β-lactam antibiotics, which inhibit the biosynthesis of bacterial cell wall, are the most widely available antibiotics used to treat a number of bacterial infections. Resistance to β-lactam antibiotics, however, has become a worldwide health care problem. Production of β-lactamases is a major and threatening resistance mechanism toward β-lactam antibiotics. Epidemiological work by Chuma et al. (2013) demonstrated a recent emergence of β-lactamase-mediated cefotaxime resistance in *Salmonella enetrica* Serovar Infantis. To counteract β-lactam resistance in pathogenic bacteria, extensive research in the past three decades has focused on the discovery of novel compounds inhibiting the β-lactamase function. Watkins et al. (2013) reviewed the novel β-lactamase inhibitors that are close to being introduced in the clinical practice. Despite the successful development of β-lactamase inhibitors for the combination therapy, the use of β-lactamase inhibitors is still challenged by the variable affinity of inhibitors to different β-lactamases and by the vast quantity of β-lactamases produced by the resistant strains. To address this issue and optimize the existing β-lactam-based therapy, Zeng and Lin (2013) proposed to inhibit the induction of β-lactamases by targeting the key players required for β-lactamase induction, such as lytic transglycosylase.

Aminoglycosides are another class of clinically important antibiotics for treating various bacterial pathogens. The increasing resistance of clinical isolates against aminoglycosides, however, has compromised the effectiveness of this class of antibiotics. A major mechanism of aminoglycoside resistance is the production of aminoglycoside-modifying enzymes. Two enzymes with aminoglycoside-modifying activities are discussed in this research topic. Shi et al. (2013) provided a comprehensive overview of the structure of aminoglycoside kinase and reported on the recent progress in the discovery of aminoglycoside phosphotransferase inhibitors using structure-guided strategies. Aminoglycoside 6′-*N*-acetyltransferase type Ib is another clinically important enzyme prevalent in a wide variety of Gram-negative pathogens. Ramirez et al. (2013) reviewed the unique sequence, genomics and functional features of this type of aminoglycoside-modifying enzymes. They also provided an insightful discussion on the development of innovative antisense technologies to combat this type of aminoglycoside resistance.

Several articles are focused on the discovery of innovative antimicrobials by harnessing our knowledge in bacterial physiology and pathogenesis. Quorum sensing is a unique cell-to-cell communication that modulates the expression of antibiotic resistance as well as virulence genes. Thus, the key compounds mediating quorum sensing such as acylated homoserine lactone have been attractive targets for antimicrobial chemotherapy. Hirakawa and Tomita (2013) reviewed recent progress in the discovery of acylated homoserine lactone inhibitors/modulators and discussed the feasibility of targeting other molecular components involved in signal transduction (e.g., regulatory elements) to modulate quorum sensing. Olivares et al. (2013) provided a timely review to analyze recent works on the intrinsic resistome, that is the concerted activity of elements required for the intrinsic resistance in *E. coli* and *P. aeruginosa*. The feasibility of using intrinsic resistome inhibitors for potentiating the effects of clinical drugs is also discussed in this review. Iino et al. (2013) used a large-scale femtoliter droplet array for single-cell analysis to assess the heterogeneity among the individual cells, enabling the identification of the novel or unconventional mechanisms of antibiotic resistance or resistance against novel antibiotics.

This research topic also includes a panel of articles focused on specific resistance mechanisms in different pathogens. Tuberculosis (TB) is notoriously known for its resistance to multiple drugs. Green and Garneau-Tsodikova (2013) focused on various mechanisms of resistance to the currently available anti-TB drugs and provided perspectives for novel strategies and lead scaffolds/compounds that are aimed at deterring these resistance mechanisms. In an effort to reduce emerging resistance, combinations of small molecules and a high-throughput synergy screening have been also explored (Zhang et al., 2007). Ilina et al. (2013) observed that a mutation in the ribosomal protein S5 is responsible for the resistance of *Neisseria gonorrhoeae* strains against multiple drugs including the decreased susceptibility to spectinomycin, cefixime and ceftriaxone. Zaheer et al. (2013) found that both therapeutic and subtherapeutic macrolide administration significantly increased the proportion of erythromycin resistant enterococci but had no effect on the development of macrolide resistance in *Mannheimia haemolytica*, both isolated from the nasopharynx. Sun et al. (2013) reported that sterol C-22 desaturase ERG5, which is highly conserved among various fungal species, is involved in azole resistance and may serve as a novel target for antifungal drugs, in particular against *Neurospora crassa* and *Fusarium verticillioides*. Using the population-based multivariate analysis, Abbes et al. (2013) observed that fluconazole resistance in *Candida glabrata* involves complex interactions between drug resistance gene expression and/or copy number.

Recent metagenomics and functional genomics studies have provided a compelling evidence that antibiotic resistance genes are widespread and the natural reservoirs of potential antibiotic resistance include many ecosystems such as in agriculture (e.g., animal manure, soil, water, wastewater lagoons), the gut of humans and food animals, and even ancient soils. The diverse range of novel antibiotic resistance genes could be accessible to clinically relevant bacteria and play a critical role in the emergence of antibiotic resistance among pathogens. Pehrsson et al. (2013) provided an insightful review for the novel resistance functions uncovered using the functional metagenomic examination of various resistance reservoirs. Municipal biosolids that are produced during the activated sludge treatment are also a significant reservoir of antibiotic resistance as assessed by Kaplan et al. (2013). Burch et al. (2013) explored an alternative approach, aerobic digestion, to reduce the quantity of antibiotic resistance genes in wastewater solids. The occurrence of antibiotics resistance genes in finfish aquaculture environments is further discussed by Miranda et al. (2013). The presence of antibiotic resistance genes in the aquatic environment is also demonstrated by Fahrenfeld et al. (2013) and Suzuki et al. (2013). To address the key issue concerning the role of environmental resistance gene reservoir in the emergence of clinically important resistant pathogens, Perry and Wright (2013) reviewed recent works suggesting genetic exchange between the environmental and clinical resistomes. Community-acquired methicillin-resistant *Staphylococcus aureus* (MRSA) has emerged as a major cause of disease in the general population. Roberts et al. (2013) examined 55 environmental MRSA isolates from 805 samples and found that 98% of them are also resistant to other classes of antibiotics in addition to methicillin, thus most likely representing the antibiotic resistance gene pool in the environment. Clearly, with the aid of high-throughput sequencing and metagenomics approaches, recent studies of natural antibiotic resistance gene reservoirs have revealed a much higher level of diversity and novelty than anticipated. However, there is still a significant knowledge gap regarding the mechanisms of horizontal gene transfer that are involved in the exchange of genes among different ecological compartments. A better understanding of these *in situ* processes is required in order to control the development, transmission, and evolution of antibiotic resistant genes.

In summary, the articles within this eBook address various timely issues related to antibiotic resistance mechanisms. Clearly, discovery of new antimicrobials as well as finding strategies to expand the useful life of existing antibiotics is important to combat the ever-increasing antimicrobial resistance. Bacteria, however, possess an enormous diversity of genes that allow them, sooner or later, to counteract the action of newly invented antibiotics. As reflected in many articles in this eBook, the natural resistomes are common and exist in diverse environmental niches. Misuse of antibiotics, in terms of application and dosage, is an additional contributing factor for the development of antibiotic resistance (Nosanchuk et al., 2014). Consequently, to mitigate antibiotic resistance, we should cautiously use antibiotics from a One Health perspective (http://www.onehealthinitiative.com/). On the other hand, as it is also reflected in this Research Topic, in parallel to the development of new of antibiotics, it is imperative to study the molecular basis of resistance development so that we can prevent and overcome antibiotic resistance by targeting resistance mechanisms, which will make the existing and novel antibiotics more effective and sustainable.

## Conflict of interest statement

The authors declare that the research was conducted in the absence of any commercial or financial relationships that could be construed as a potential conflict of interest.

## References

[B1] Abbes S. Mary C. Sellami H. Michel-Nguyen A. Ayadi A. Ranque S. (2013). Interactions between copy number and expression level of genes involved in fluconazole resistance in *Candida glabrata*. Front. Cell. Infect. Microbiol. 3:74. 10.3389/fcimb.2013.00074 24273749 PMC3822285

[B2] Baucheron S. Monchaux I. Le H. S. Weill F. X. Cloeckaert A. (2014). Lack of efflux mediated quinolone resistance in *Salmonella enterica* serovars Typhi and Paratyphi A. Front. Microbiol. 5:12. 10.3389/fmicb.2014.00012 24478769 PMC3902205

[B3] Burch T. R. Sadowsky M. J. Lapara T. M. (2013). Aerobic digestion reduces the quantity of antibiotic resistance genes in residual municipal wastewater solids. Front. Microbiol. 4:17. 10.3389/fmicb.2013.00017 23407455 PMC3569665

[B4] Chuma T. Miyasako D. Dahshan H. Takayama T. Nakamoto Y. Shahada F.. (2013). Chronological change of resistance to beta-Lactams in *Salmonella enterica* serovar infantis isolated from broilers in Japan. Front. Microbiol. 4:113. 10.3389/fmicb.2013.00113 23734146 PMC3659313

[B5] Deng Z. Shan Y. Pan Q. Gao X. Yan A. (2013). Anaerobic expression of the*gadE-mdtEF* multidrug efflux operon is primarily regulated by the two-component system ArcBA through antagonizing the H-NS mediated repression. Front. Microbiol. 4:194. 10.3389/fmicb.2013.00194 23874328 PMC3708157

[B6] Fahrenfeld N. Ma Y. O'Brien M. Pruden A. (2013). Reclaimed water as a reservoir of antibiotic resistance genes: distribution system and irrigation implications. Front. Microbiol. 4:130. 10.3389/fmicb.2013.00130 23755046 PMC3664959

[B7] Green K. D. Garneau-Tsodikova S. (2013). Resistance in tuberculosis: what do we know and where can we go? Front. Microbiol. 4:208. 10.3389/fmicb.2013.00208 23888158 PMC3719028

[B8] Hirakawa H. Tomita H. (2013). Interference of bacterial cell-to-cell communication: a new concept of antimicrobial chemotherapy breaks antibiotic resistance. Front. Microbiol. 4:114. 10.3389/fmicb.2013.00114 23720655 PMC3652290

[B9] Iino R. Matsumoto Y. Nishino K. Yamaguchi A. Noji H. (2013). Design of a large-scale femtoliter droplet array for single-cell analysis of drug-tolerant and drug-resistant bacteria. Front. Microbiol. 4:300. 10.3389/fmicb.2013.00300 24109478 PMC3790107

[B10] Ilina E. N. Malakhova M. V. Bodoev I. N. Oparina N. Y. Filimonova A. V. Govorun V. M. (2013). Mutation in ribosomal protein S5 leads to spectinomycin resistance in *Neisseria gonorrhoeae*. Front. Microbiol. 4:186. 10.3389/fmicb.2013.00186 23847609 PMC3706878

[B11] Kaplan E. Ofek M. Jurkevitch E. Cytryn E. (2013). Characterization of fluoroquinolone resistance and qnr diversity in Enterobacteriaceae from municipal biosolids. Front. Microbiol. 4:144. 10.3389/fmicb.2013.00144 23781217 PMC3678080

[B12] Miranda C. D. Tello A. Keen P. L. (2013). Mechanisms of antimicrobial resistance in finfish aquaculture environments. Front. Microbiol. 4:233. 10.3389/fmicb.2013.00233 23986749 PMC3749489

[B13] Morita Y. Tomida J. Kawamura Y. (2012). MexXY multidrug efflux system of *Pseudomonas aeruginosa*. Front. Microbiol. 3:408. 10.3389/fmicb.2012.00408 23233851 PMC3516279

[B14] Nosanchuk J. D. Lin J. Hunter R. P. Aminov R. I. (2014). Low-dose antibiotics: current status and outlook for the future. Front. Microbiol. 5:478. 10.3389/fmicb.2014.00478 25309518 PMC4159977

[B15] Olivares J. Bernardini A. Garcia-Leon G. Corona F. Sanchez B. Martinez J. L. (2013). The intrinsic resistome of bacterial pathogens. Front. Microbiol. 4:103. 10.3389/fmicb.2013.00103 23641241 PMC3639378

[B16] Pehrsson E. C. Forsberg K. J. Gibson M. K. Ahmadi S. Dantas G. (2013). Novel resistance functions uncovered using functional metagenomic investigations of resistance reservoirs. Front. Microbiol. 4:145. 10.3389/fmicb.2013.00145 23760651 PMC3675766

[B17] Perry J. A. Wright G. D. (2013). The antibiotic resistance “mobilome”: searching for the link between environment and clinic. Front. Microbiol. 4:138. 10.3389/fmicb.2013.00138 23755047 PMC3667243

[B18] Ramirez M. S. Nikolaidis N. Tolmasky M. E. (2013). Rise and dissemination of aminoglycoside resistance: the aac(6′)-Ib paradigm. Front. Microbiol. 4:121. 10.3389/fmicb.2013.00121 23730301 PMC3656343

[B19] Roberts M. C. Soge O. O. No D. (2013). Comparison of multi-drug resistant environmental methicillin-resistant *Staphylococcus aureus* isolated from recreational beaches and high touch surfaces in built environments. Front. Microbiol. 4:74. 10.3389/fmicb.2013.00074 23577006 PMC3616243

[B20] Shi K. Caldwell S. J. Fong D. H. Berghuis A. M. (2013). Prospects for circumventing aminoglycoside kinase mediated antibiotic resistance. Front. Cell. Infect. Microbiol. 3:22. 10.3389/fcimb.2013.00022 23805415 PMC3691515

[B21] Sun X. Wang W. Wang K. Yu X. Liu J. Zhou F.. (2013). Sterol C-22 desaturase ERG5 mediates the sensitivity to antifungal azoles in *Neurospora crassa* and *Fusarium verticillioides*. Front. Microbiol. 4:127. 10.3389/fmicb.2013.00127 23755044 PMC3666115

[B22] Suzuki S. Ogo M. Miller T. W. Shimizu A. Takada H. Siringan M. A. (2013). Who possesses drug resistance genes in the aquatic environment?: sulfamethoxazole (SMX) resistance genes among the bacterial community in water environment of Metro-Manila, Philippines. Front. Microbiol. 4:102. 10.3389/fmicb.2013.00102 23641240 PMC3639423

[B23] Usui M. Nagai H. Hiki M. Tamura Y. Asai T. (2013). Effect of antimicrobial exposure on AcrAB Expression in *Salmonella enterica* subspecies enterica serovar choleraesuis. Front. Microbiol. 4:53. 10.3389/fmicb.2013.00053 23503095 PMC3596762

[B24] Watkins R. R. Papp-Wallace K. M. Drawz S. M. Bonomo R. A. (2013). Novel beta-lactamase inhibitors: a therapeutic hope against the scourge of multidrug resistance. Front. Microbiol. 4:392. 10.3389/fmicb.2013.00392 24399995 PMC3871716

[B25] Zaheer R. Cook S. R. Klima C. L. Stanford K. Alexander T. Topp E.. (2013). Effect of subtherapeutic vs. therapeutic administration of macrolides on antimicrobial resistance in *Mannheimia haemolytica* and enterococci isolated from beef cattle. Front. Microbiol. 4:133. 10.3389/fmicb.2013.00133 23750157 PMC3664329

[B26] Zeng X. Lin J. (2013). Beta-lactamase induction and cell wall metabolism in Gram-negative bacteria. Front. Microbiol. 4:128. 10.3389/fmicb.2013.00128 23734147 PMC3660660

[B27] Zhang L. Yan K. Zhang Y. Bian J. Zheng C. Sun H.. (2007). High-throughput synergy screening identifies microbial metabolites as combination agents for the treatment of fungal infections. Proc. Natl. Acad. Sci. U.S.A. 104, 4606–4611. 10.1073/pnas.0609370104 17360571 PMC1838648

